# Effect of combination of multiple anti-inflammatory drugs strategy on postoperative delirium among older patients undergoing hip fracture surgery: a pilot randomized controlled trial

**DOI:** 10.1186/s12916-025-03946-x

**Published:** 2025-02-21

**Authors:** Ayixia Nawan, Zilong Wu, Bailin Jiang, Geng Wang, Wenchao Zhang, Yi Feng

**Affiliations:** 1 Department of Anesthesiology, Peking University People’s Hospital, No.11 Xizhimen South Street, Xicheng District, Beijing, 100044 China; 2 Department of Anesthesiology, Beijing Jishuitan Hospital, Capital Medical University, Beijing, China; 3 Department of Pain Medicine, Peking University People’s Hospital, Beijing, China; 4 Key Laboratory for Neuroscience, Ministry of Education, National Health Commission, Peking University, Beijing, China

**Keywords:** Aged, Anti-inflammatory, Hip fracture, Perioperative management, Pharmacological interventions, Postoperative delirium

## Abstract

**Background:**

Postoperative delirium is the most common complication in older patients and is associated with surgery-induced inflammation. Although inflammation plays a key role in delirium, the potential benefits of a comprehensive anti-inflammatory approach to managing perioperative systemic inflammation remain underexplored. This study evaluated whether a perioperative anti-inflammatory bundle strategy, combining dexmedetomidine, glucocorticoids, ulinastatin, and nonsteroidal anti-inflammatory drugs, reduces the risk of postoperative delirium in older patients undergoing hip fracture surgery.

**Methods:**

This dual-center, double-blind, placebo-controlled, parallel-group, pilot study was conducted from August 2023 to January 2024 at two tertiary university hospitals. A total of 132 patients aged ≥ 65 years with an American Society of Anesthesiologists physical status of 2 or 3 scheduled for elective hip fracture surgery were screened and randomized to receive either an anti-inflammatory drug bundle or a placebo. The primary outcome was postoperative delirium, identified within the first three postoperative days. Postoperative blood inflammatory markers and acute pain were measured for mediation analysis.

**Results:**

Of the 132 patients randomized, 123 (93%) completed the trial (mean age, 82 years; 75% women). The prevalence of postoperative delirium was significantly lower in the anti-inflammatory bundle group (15%, 9/62) compared to the placebo group (44%, 27/61) (risk difference, − 30 percentage points [95% CI, − 45 to − 15]; relative risk [RR], 0.33 [95% CI, 0.17 to 0.64]; *P* = 0.001). No major adverse events were reported in either group. The postoperative CRP level in the anti-inflammatory bundle group was significantly lower (predicted mean difference: − 29.4 [95% CI: − 46.5, − 12.2] mg·L^−1^; adjusted *P* < 0.001). Mediation analysis showed a significant indirect association between the anti-inflammatory bundle and postoperative delirium through reduced systemic inflammation (odds ratio [OR], 0.61 [95% CI, 0.26 to 0.87]).

**Conclusions:**

This study demonstrates that a perioperative anti-inflammatory bundle significantly reduces the prevalence of postoperative delirium in older patients undergoing hip fracture surgery, without major side effects. Systemic inflammation mediates the protective effect of the intervention. These findings provide preliminary evidence supporting the anti-inflammatory bundle strategy, paving the way for large-scale multicenter trials to optimize postoperative delirium prevention strategies.

**Trial registration:**

This study was registered in the Chinese Clinical Trial Registry (ChiCTR2300074303) by Ayixia Nawan on August 3, 2023, prior to patient enrollment.

**Supplementary Information:**

The online version contains supplementary material available at 10.1186/s12916-025-03946-x.

## Background

Postoperative delirium (POD) is the most common complication of surgery among older patients [1], with an incidence of approximately 50% in those undergoing hip fracture surgeries [2–4]. POD is linked to cognitive decline, dementia, functional deterioration, and increased mortality [1, 5, 6], with healthcare costs reaching $44,291 per patient per year [7]. Given its preventable nature [7, 8], effective strategies are needed, especially for older patients undergoing hip fracture surgeries.

However, effective strategies to prevent POD remain lacking. Current prevention methods [9], such as avoiding benzodiazepines, titrating anesthetic depth, and managing pain, are often passive, controversial, and inconsistently effective [3, 10–13]. Although multidisciplinary non-pharmacologic interventions show promise [2, 8, 14], their complexity and variability hinder widespread adoption [9, 15]. Current pharmacological interventions remain largely focused on pain control, with limited attention to broader inflammatory mechanisms contributing to POD [8, 16].

Surgery-evoked systemic inflammation is a key contributor to POD development [17], with accumulating evidence suggesting that suppressing inflammation may reduce its incidence [18–21]. Perioperative drugs such as dexmedetomidine [8, 19, 22–24], glucocorticoids [20, 21], ulinastatin [25], and nonsteroidal anti-inflammatory drugs (NSAIDs) [26, 27] have demonstrated anti-inflammatory effects and potential for POD prevention. However, their effectiveness when used individually has been suboptimal, and comprehensive pharmacological approaches targeting systemic inflammation remain underexplored [28]. Combining these drugs may enhance their anti-inflammatory effects, providing a more holistic strategy to mitigate perioperative inflammation and potentially prevent POD.

Given the complexity and potential risks associated with multidrug regimens, it is critical to conduct a pilot study to assess feasibility and gather preliminary evidence of efficacy. This will lay the groundwork for larger multicenter trials to develop and validate new clinical strategies for POD prevention. In this pilot study, we introduced a perioperative anti-inflammatory bundle strategy combining multiple anti-inflammatory drugs. We hypothesized that this strategy could reduce POD incidence in older patients undergoing hip fracture surgery by mitigating systemic inflammation. Furthermore, we examined the mediation effect of systemic inflammation on the relationship between the anti-inflammatory bundle and POD.

## Methods

### Study design

This dual-center, double-blind, placebo-controlled, parallel-group study was conducted at two class A tertiary comprehensive hospitals in China. Ethical approval (Approval No.: K2023-139–00) was granted by the Ethics Committee of Beijing Jishuitan Hospital, Beijing, China on May 23, 2023. Written informed consent was obtained from all patients or their legal surrogates. The study was registered in the Chinese Clinical Trial Registry (ChiCTR2300074303) by Ayixia Nawan on August 3, 2023, prior to patient enrollment. This manuscript adheres to the Consolidated Standards of Reporting Trials (CONSORT) guidelines.

### Participants

Eligible participants were patients aged 65 years or older, classified as American Society of Anesthesiologists (ASA) physical status 2 or 3, and scheduled for hip fracture surgery. To reduce potential bias from circadian rhythms and ensure adequate intervals between delirium assessments on the day of surgery, only patients whose surgeries were completed in the morning were included. Exclusion criteria included (a) refusal to participate, (b) history of long-term opioid use or alcohol abuse, (c) inability to assess cognitive function, (d) contraindications to study drugs, and (e) cancelations or changes in scheduled surgeries, including intraoperative events leading to significant alterations in the course or duration of the surgery.

### Randomization and blinding

The participants were randomly allocated to receive either an anti-inflammatory drug bundle (anti-inflammatory bundle group) or a placebo (control group) in a 1:1 ratio, with randomization stratified by center. A research assistant generated and securely stored the randomization, keeping allocations concealed from participants and investigators until the study’s conclusion. An independent pharmacist team, aware of the group assignments, prepared the trial drugs or placebos before surgery. Patients, anesthesiologists, and outcome assessors were blinded to the group assignments.

### Perioperative management and interventions

Patients underwent total hip arthroplasty (THA), hemiarthroplasty, cannulated screw fixation, or proximal femoral nail anti-rotation (PFNA) surgeries at the discretion of the surgeon. All surgeries were performed under routine spinal anesthesia, supplemented with ultrasound-guided fascia iliaca compartment block analgesia. Postoperatively, all participants received patient-controlled intravenous analgesia with sufentanil. Anesthetic techniques and hemodynamic management adhered to local practices, with the use of anticholinergic medications, benzodiazepines, or ketamine prohibited.

In the anti-inflammatory bundle group, patients received perioperative intravenous anti-inflammatory drugs, including (a) a single preoperative dose of dexamethasone (0.1 mg·kg^−1^); (b) a single preoperative dose of ulinastatin (10,000 U·kg^−1^); (c) continuous intraoperative and postoperative infusion of dexmedetomidine (0.2–0.5 μg·kg^−1^·h^−1^ until the end of surgery and 0.03 μg·kg^−1^·h^−1^ for 48 h postoperatively); and (d) a single postoperative dose of flurbiprofen axetil (50 mg) followed by continuous infusion (0.06 mg·kg^−1^·h^−1^) for 48 h postoperatively. Dosages and administration methods were determined according to routine clinical practice, with adjustments towards the lower end of the standard range to minimize potential adverse events.

The control group received 0.9% saline and 0.05% lipid emulsion (to mimic flurbiprofen axetil) as a placebo. Pharmacists prepared all trial drugs to ensure that anesthesiologists were blinded to group assignments.

### Assessment and outcomes

The primary outcome of this study was POD, defined as delirium identified during the day of surgery (postoperative day 0) and the first 2 days after surgery [28]. Delirium was identified using the confusion assessment method (CAM). A team of geriatricians, blinded to the group assignments, conducted assessments twice daily on the day of surgery (post-anesthesia care unit and afternoon) and postoperative days 1 and 2 (morning and afternoon). For patients who experienced POD, the Richmond Agitation-Sedation Scale (RASS) and Delirium Rating Scale-Revised-98 (DRS-R-98) severity scale [29] were used to assess the motor subtypes and severity of delirium, respectively.

Secondary outcomes included postoperative pain at rest and during activity and the length of hospital stay. A blinded nurse anesthetist team assessed acute pain using the numeric rating scale (NRS) at 12-h intervals during the first 48 h postoperatively [30]. The time-weighted average NRS [16] was calculated for the analysis.

Major adverse events included postoperative acute myocardial infarctions, strokes, gastrointestinal ulcers, acute renal failure, and hypotensive shock.

Baseline clinical data, including ASA physical status, type of fracture and surgery, preoperative pain, preoperative opioid use, anxiety, depression, cognitive function, frailty status, preoperative hemoglobin level, and preoperative cerebrospinal fluid (CSF) lactate concentration, were collected on the day before operation to evaluate their association with POD. Anxiety and depression were assessed using the Hospital Anxiety and Depression Scale (HADS, consisting of two subscales for estimating anxiety [HADS-A] and depression [HADS-D] and both ranging between 0 and 21 points, where HADS-A ≥ 8 indicates anxiety and HADS-D ≥ 8 indicates depression) [31]. Cognitive function was evaluated using the Montreal Cognitive Assessment (MoCA, ranging between 0 and 30 points, 30 points for best performance). Frailty status was assessed using the FRAIL scale (0 to 5 points, where 0 indicates robust status and ≥ 3 indicates frail status). CSF was attained during spinal anesthesia. Postoperative data that may affect the primary outcomes were recorded to inspect any discrepancies that may still exist after randomization, including intensive care unit (ICU) admission, postoperative rescue opioid use, and postoperative hemoglobin level.

Perioperative blood levels of inflammatory markers, including S100 calcium-binding protein β (S100β), neuron-specific enolase (NSE), and C-reactive protein (CRP), were measured preoperatively and on the operative day and postoperative day 1. For CRP, only the values on postoperative day 1 were analyzed because of missing data on the operative day.

### Sample size calculation

According to our previous study on a comparable patient population [32], a sample size of 118 patients (59 in each group) was determined based on the assumption that 45% would experience POD and that the anti-inflammatory bundle strategy would reduce the incidence to 20%. It provided 85% power at a 2-sided *α* level of 0.05 to detect differences. Considering a dropout rate of approximately 10%, we planned to enroll 132 patients (66 patients in each group).

### Statistical analysis and mediation analysis

Patients were analyzed according to the randomization group. Multiple imputations were planned for missing data. Statistical analyses included the *t*-test or Mann–Whitney *U* test for continuous variables, and the chi-square test or Fisher’s exact test for categorical data. A modified Poisson regression model was used to adjust for the primary outcomes. A subgroup analysis was conducted for the population that developed POD. Sensitivity analyses were established a priori and performed using an alternate definition of the primary outcome which was met if a CAM-identified POD was combined with a DRS-R-98 score greater than 15 [29]. The between-group difference in perioperative inflammatory markers was compared using a two-way analysis of variance (ANOVA) and Sidak’s test.

Least absolute shrinkage and selection operator (LASSO) and logistic regression analyses were used to identify predictors of POD. Parameters related to postoperative pain and inflammatory markers that entered into the final model for predicting POD were used as mediators in mediation analysis. Mediation analysis was conducted using PROCESS (version 4.2 beta; Andrew F. Hayes). Bootstrapping (5000 iterations) was used to estimate the confidence intervals of the indirect effects. Given that the sample size calculation was based on the primary outcome, it may not have provided sufficient power for developing a predictive model or conducting mediation analysis. Therefore, the predictors for POD and the subsequent mediation analysis should be considered preliminary and exploratory.

Statistical significance was set at *P* < 0.05. IBM SPSS Statistics (version 25.0; IBM Corp., Armonk, NY, USA), Prism (version 8.3.0; GraphPad Software, LLC, Boston, MA, USA), and R software (version 4.2.0; R Foundation for Statistical Computing, Vienna, Austria; https://www.R-project.org) were used for statistical analyses.

## Results

### Patients

**Fig. 1:**
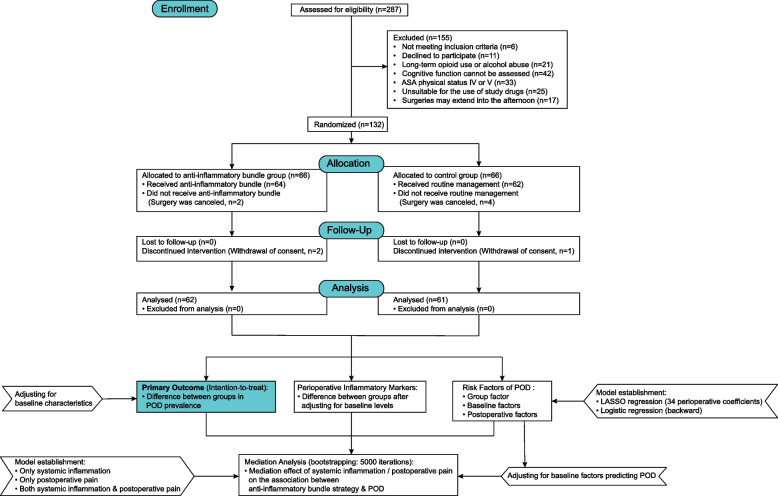
Flow of participants through the trial and statistical analysis plan. ASA, American Society of Anesthesiologists; POD, postoperative delirium; LASSO, least absolute shrinkage and selection operator

A total of 287 patients were screened for eligibility between August 2023 and January 2024, of whom 132 were randomized. All patients received the assigned intervention, except for six patients whose surgeries were canceled after randomization. During the follow-up period, three patients withdrew consent. Therefore, the final analysis included 123 patients (93%; 62 in the anti-inflammatory bundle group and 61 in the control group) (Fig. 1). No data were missing. The mean age of the patients was 82 ± 7 years, and 92 (75%) were female. Baseline characteristics were comparable between the two groups (Table 1).

**Table 1:** Baseline data of patients

Characteristic	Anti-inflammatory bundle (*n* = 62)	Control group (*n* = 61)
Age (year)	84 ± 6	80 ± 7
Male [*n* (%)]	14 (23)	17 (28)
BMI (kg m^−2^)	23 ± 4	24 ± 3
ASA grade of III or severer [*n* (%)]	58 (94)	55 (90)
Type of fracture [*n* (%)]		
Femoral neck fractures	52 (84)	42 (69)
Intertrochanteric fractures	10 (16)	19 (31)
Type of surgery [*n* (%)]		
Hemiarthroplasty	50 (81)	41 (67)
PFNA	11 (18)	19 (31)
THA	1 (2)	1 (2)
Pain at rest (NRS)	3 [2, 4]	3 [2, 4]
Pain on activity (NRS)	6 [5, 6]	6 [5, 6]
Preoperative opioids [*n* (%)]	30 (48)	33 (54)
Anxiety [*n* (%)]	27 (44)	26 (43)
Depression [*n* (%)]	18 (29)	11 (18)
MoCA	26 [25, 27]	26 [25, 27]
FRAIL score	3 [3, 4]	3 [2, 3]
Hemoglobin (g L^−1^)	113 ± 15	109 ± 19
CSF lactate (mmol L^−1^)	1.4 [1.0, 2.3]	0.9 [0.7, 1.3]
S100β (μg L^−1^)	0.6 [0.5, 0.8]	0.7 [0.5, 1.0]
NSE (ng ml^−1^)	11 [9, 15]	11 [10, 13]
CRP (mg L^−1^)	9 [ 2, 42]	18 [3, 60]

Continuous variables are expressed as mean ± SD or median [25th, 75th percentiles], as appropriate. Categorical data are presented as the number of events (proportion).

*BMI *body mass index; *ASA *American Society of Anesthesiologists, *PFNA *proximal femoral nail anti-rotation, *THA *total hip arthroplasty, *NRS *numeric rating scale, *MoCA *Montreal Cognitive Assessment, *CSF *cerebrospinal fluid, *NSE *neuron-specific enolase, *CRP *C-reactive protein

### Primary outcome

The occurrence of delirium within the first 48 h postoperatively was 15% (9/62) in the anti-inflammatory bundle group and 44% (27/61) in the control group, resulting in a risk difference of − 30 percentage points (95% CI, − 45 to − 15). The anti-inflammatory bundle strategy significantly reduced the risk of POD (RR, 0.33 [95% CI, 0.17 to 0.64]; *P* = 0.001).

**Fig. 2:**
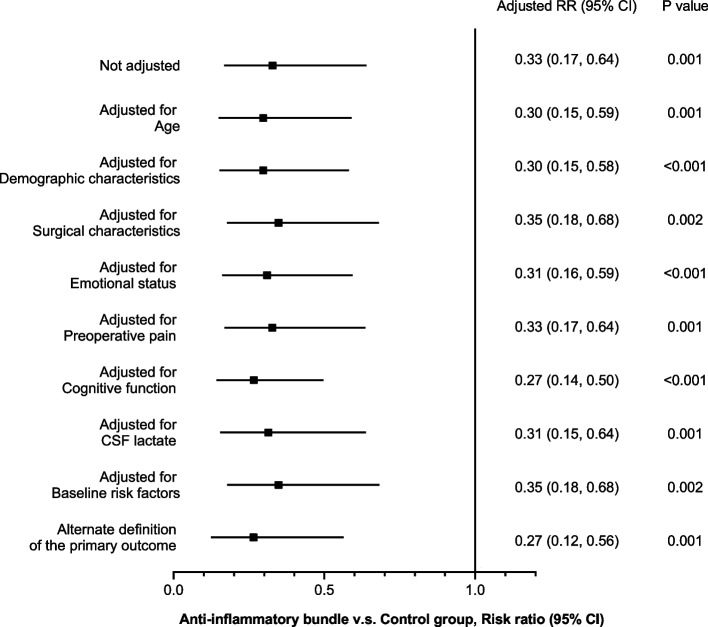
Adjusted primary outcome and sensitivity analysis. Patient characteristics included age, sex, and body mass index. Surgical characteristics included type of fracture and surgery. Emotional status encompasses anxiety and depression. Preoperative pain was assessed at rest and during activities. Cognitive function was evaluated using the Montreal Cognitive Assessment (MoCA). Baseline risk factors included MoCA scores, baseline C-reactive protein values, and baseline hemoglobin levels. Sensitivity analysis was performed using an alternate definition of the primary outcome which was met if a CAM-identified POD was combined with a DRS-R-98 score greater than 15. CSF, cerebrospinal fluid; POD, postoperative delirium; RR, risk ratio; CAM, confusion assessment method

Adjusting for baseline characteristics that could be associated with POD did not change the preventive effect on the primary outcome. The finding was consistent in the sensitivity analysis (Fig. 2, Table 2).

**Table 2:** Outcomes and postoperative data

Outcomes	Anti-inflammatory bundle (*n* = 62)	Control group (*n* = 61)	Risk/median difference^a^ [(95% CI), %]	Risk ratio (95% CI)	*P* value
**Primary outcome**					
POD [*n* (%)]	9 (14.5)	27 (44.3)	− 29.7 (− 45.0, − 14.5)	0.33 (0.17, 0.64)	0.001
**Secondary outcomes**					
Pain at rest (NRS)	1.0 [1.0, 1.0]	2.0 [1.0, 2.0]	− 1.0 (− 1.0, − 0.5)	–	< 0.001
Pain on activity (NRS)	3.0 [3.0, 4.0]	4.0 [3.2, 4.0]	− 1.0 (− 1.0, − 0.2)	–	< 0.001
Length of hospital stay (day)	4 [4, 5]	5 [4, 6]	0 [− 1, 0]	–	0.075
**Postoperative data that may affect primary outcome**					
ICU admission [*n* (%)]	9 (14.5)	8 (13.1)	1.4 (− 10.8, 13.6)	1.11 (0.46, 2.68)	1.000
Postoperative opioids [*n* (%)]	5 (8.1)	11 (18.0)	− 10.0 (− 21.8, 1.8)	0.45 (0.17, 1.21)	0.115
Hemoglobin (g L^−1^)	108 [100, 115]	107 [96, 115]	2 (− 3, 7)	–	0.275

*CI *confidence interval, *POD *postoperative delirium, *NRS *numeric rating scale, *ICU *intensive care unit

^a^Pseudo-median difference was calculated using the Hodges-Lehmann estimate

In the subgroup of patients who experienced POD, the severity score in the anti-inflammatory bundle group was lower than that in the control group (25 [18, 33] vs. 30 [30, 35]; median difference, − 6 [95% CI, − 122 to − 0]; *P* = 0.045). There was no difference in the motor subtypes of delirium between the two groups (Additional file 1: Supplemental Table 1).

### Secondary outcomes

The patients in the anti-inflammatory bundle group had lower pain scores at rest (1 [1, 1] vs. 2 [1, 2]; median difference, −1.0 [95% CI, −1.0 to −0.5]) and activity (3 [3, 4] vs. 4 [3, 4]; median difference, −1.0 [95% CI, −1.0 to −0.2]) than those in the control group (Table 2). There was no significant difference between groups regarding length of hospital stay and postoperative characteristics (ICU admission, opioid administration, and hemoglobin level; Table 2). No major adverse events were reported in either of the groups.

### Perioperative inflammatory markers

**Fig. 3:**
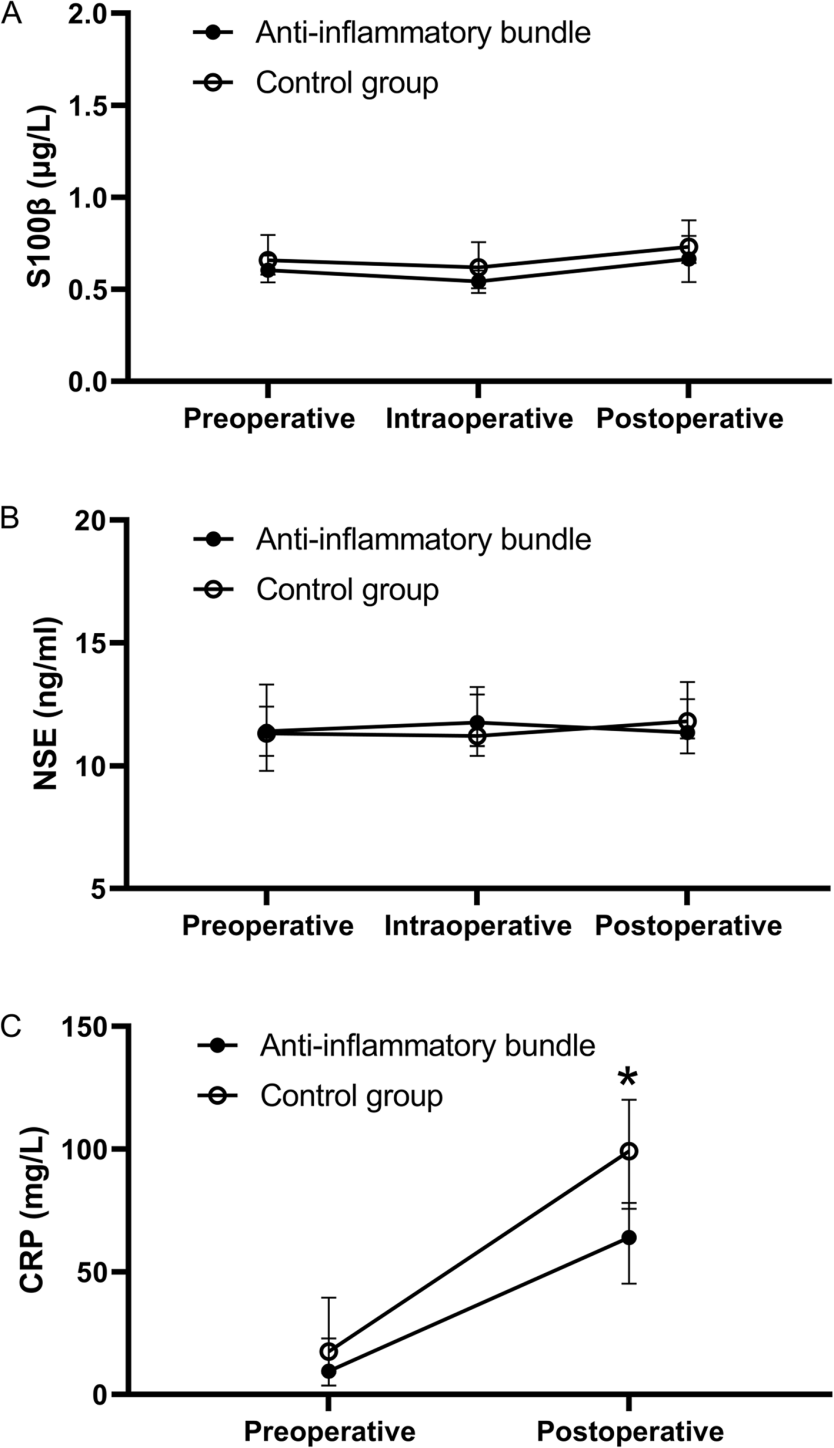
Perioperative blood levels of inflammatory markers. The data are presented as the median with a 95% confidence interval (CI) and were analyzed using two-way analysis of variance (ANOVA) (with Sidak’s multiple comparisons test when appropriate). **A** There was no significant difference in S100β levels between groups (*F* = 0.49, *P* = 0.483). **B** There was no significant difference in NSE levels between groups (*F* = 1.01, *P* = 0.318). **C** Postoperative CRP levels in the anti-inflammatory bundle group were significantly lower than those in the control group (predicted mean difference: − 29.4 [95% CI: − 46.5, − 12.2] mg·L.^−1^, adjusted *P* < 0.001)

There were no significant differences in S100β and NSE levels between groups (Fig. 3). The postoperative CRP level in the anti-inflammatory bundle group was significantly lower than that in the control group (predicted mean difference: − 29.4 [95% CI: − 46.5, − 12.2] mg·L^−1^; adjusted *P* < 0.001; Fig. 3).

### Factors significantly associated with POD

Based on one standard error criterion in the preliminary LASSO regression model, six potential predictors were chosen out of 34 coefficients (Additional file 2: Fig. S1). All six candidates entered the final logistic regression model and were significantly associated with the occurrence of POD (Additional file 1: Supplemental Table 2). These factors included (a) group factor: anti-inflammatory management bundle; (b) baseline factors: preoperative MoCA score, preoperative CRP value, and preoperative hemoglobin level; and (c) postoperative factors: postoperative pain score at rest and postoperative CRP value.

### Preliminary mediation analysis

After adjusting for baseline factors predicting POD, a significant indirect association via systemic inflammation was observed between the anti-inflammatory bundle strategy and POD (OR, 0.61 [95% CI, 0.26 to 0.87]; Additional file 2: Fig. S2). However, the indirect association between the anti-inflammatory bundle strategy and POD, mediated by postoperative pain, was not statistically significant (Additional file 1: Supplemental Table 3). In sensitivity analysis, these findings were consistent (Additional file 1: Supplemental Table 4).

## Discussion

This study introduced a perioperative anti-inflammatory bundle strategy using routinely used anti-inflammatory drugs, reducing the prevalence of POD to one-third of that in the control group without major adverse events. This significant reduction may be one of the most effective results in POD prevention [8, 12, 14, 15, 22, 23, 33]. Although the effect could be exaggerated due to the limited sample size, this approach appears promising. Further research involving more centers and patients is warranted.

According to local practice, patients were typically discharged on postoperative day 3 if no adverse events occurred. Therefore, delirium was screened during the first 3 days. Most delirium that occurs after surgery is on postop day 1 and 2. Although this approach meets the recommendation to measure POD for at least 3 days starting on the day of surgery [28], the potential underestimation of POD incidence due to the limited follow-up period should be acknowledged. Additionally, preoperative delirium was not assessed in this study. However, screening for delirium preoperatively is often impractical in routine clinical settings, and excluding patients with pre-existing delirium may cause study findings to diverge from real-world conditions. This approach aligned with most reference studies reporting POD prevalence, which similarly did not exclude patients with pre-existing delirium [12, 14, 16, 20, 23, 26, 33–35]. Therefore, this study focused on the prevalence, rather than the incidence, of delirium, which is more relevant to clinical practice. Although evaluating the effects of an anti-inflammatory strategy on patients with pre-existing delirium was beyond the scope of this study, it remains an area worthy of further investigation.

POD significantly affects quality of life and is strongly associated with increased morbidity and mortality [5, 6], especially in older patients [2, 23], with rates reaching approximately 50% in those undergoing hip fracture surgeries [2–4]. Although several strategies have been proposed to prevent POD [3, 10–13], non-pharmacological methods are currently recommended by most evidence and guidelines [2, 8, 9, 14, 15]. However, these complex approaches require multidisciplinary intervention and may result in variable implementation and inconsistent outcomes [9, 15, 33].

Pharmacological prophylaxis for preventing POD offers a pragmatic approach, yet current interventions mainly focus on pain control [8, 9, 16]. Recent literature confirms the pathogenic role of surgery-induced inflammation, highlighting the potential of anti-inflammatory strategies [28]. Most drugs in this study target postoperative inflammation and alleviate acute pain. Mediation analysis revealed that the association between the interventions and POD is mediated by systemic inflammation rather than acute pain. Both groups achieved effective pain control through fascia iliaca compartment block and patient-controlled intravenous analgesia with sufentanil [36]. Although there was a significant difference between the groups, acute pain levels remained mild in both. Therefore, the drug bundle’s impact on reducing POD should be interpreted primarily as an anti-inflammatory effect, suggesting that controlling systemic inflammation may help prevent POD and warrants further investigation.

Recent research increasingly highlights inflammation’s role in POD [28], with studies documenting changes like circulating cytokines, inflammatory transcripts in the brain, blood–brain barrier impairments, and alterations in microglial cells and astrocytes. While these inflammatory mechanisms are well-established, the broader potential of anti-inflammatory approaches remains underexplored. Combining multiple anti-inflammatory drugs might offer a more effective strategy for managing inflammation and preventing POD. Given the caution required with polypharmacy in older patients, validating the benefits of such combinations through clinical trials is essential. This study found that the combined use of dexmedetomidine, glucocorticoids, ulinastatin, and NSAIDs was significantly more effective in reducing POD prevalence (RR = 0.33). In contrast, when used individually, even the most promising drug, dexmedetomidine, showed weaker effects (RR = 0.60) [22, 34]. The superior efficacy of the combination likely stems from its ability to target various anti-inflammatory pathways, though further studies are needed to confirm these findings. Additionally, the impact of the duration of dexmedetomidine infusion warrants further investigation.

Future studies should determine the optimal drugs, dosages, and administration methods for the anti-inflammatory bundle. While this pilot study supports the concept, it does not establish the specific drug combination as the definitive formulation. Additional research is needed to identify the most effective and safe drug formulations.

Safety is a key concern with combined drug use. The drugs in this study are routinely used in perioperative practice and are generally safe for patients without contraindications. At our centers, continuous postoperative NSAID infusion has been safely implemented and recommended for years. We followed standard dosing strategies, adjusting to the lower end of the range, and observed no major adverse events. However, further studies are needed to assess detailed adverse event profiles and rates of drug side effects, particularly as new drug formulations are developed.

Anti-inflammatory treatments generally reduce both brain and peripheral inflammation. This study examined blood markers associated with POD, including S100β (a biomarker for blood–brain barrier dysfunction) [37], NSE (a marker for neuronal damage) [38], and CRP (a common systemic inflammatory marker) [39]. Only CRP showed significant postoperative changes, suggesting that S100β and NSE levels were likely already elevated preoperatively. This elevation may partly explain the high susceptibility and prevalence of POD in this population. Postoperative CRP levels were significantly lower in the anti-inflammatory bundle group, indicating that the perioperative anti-inflammatory strategy effectively controlled systemic inflammation, thereby reducing POD occurrence. Since the aim of this pilot study was to explore whether systemic inflammation or acute pain mediated the association between the interventions and POD, rather than to identify risk factors for POD, CRP levels were used as a continuous variable in the prediction model and mediation analysis. No attempts were made to establish a threshold for CRP values. The lack of postoperative elevation in S100β and NSE levels could imply the presence of preexisting neuroinflammation and blood–brain barrier dysfunction. However, the strategy may still mitigate POD risk by effectively targeting systemic inflammation under these conditions.

This study focused on older patients undergoing hip fracture surgery, but previous research on the individual drugs used here has shown consistent POD prevention across various surgeries [8, 19–23, 25–27]. Thus, the perioperative anti-inflammatory bundle strategy could potentially benefit older patients experiencing distinct inflammatory process. However, its effectiveness may vary depending on the surgery, warranting further testing with more diverse samples.

This study has some limitations. First, as a dual-center pilot study with a small sample size, the findings may overestimate the effect. The sample size was not sufficiently powered to identify predictors for POD. However, the primary aim of this pilot study was to evaluate the approach’s effectiveness in preventing POD. The sample size was calculated based on prior analyses [32], with a statistical power of 85% to detect a significant effect. Second, the study may have underestimated POD incidence due to the 3-day follow-up period. However, since the most pronounced disturbances usually occur shortly after surgery and the control group had a high prevalence of POD, the primary findings are unlikely to be significantly affected by this limitation. Finally, to reduce potential bias from circadian rhythms and ensure adequate intervals between delirium assessments on the day of surgery, only patients whose surgeries were completed in the morning were included. This limitation precluded analysis of circadian rhythm and sleep quality, factors that may be associated with POD [40], potentially restricting the applicability of the findings to situations where these concerns are relevant.

## Conclusions

This pilot study demonstrated that a perioperative anti-inflammatory bundle strategy significantly reduced the prevalence of POD by mitigating systemic inflammation, without major adverse events, in older patients undergoing hip fracture surgery. Systemic inflammation was identified as a mediator in the relationship between pharmacological intervention and POD. Larger multicenter trials are needed to confirm these findings and further investigate the role of anti-inflammatory approaches in POD prevention.

## Supplementary Information

Additional file 1. Supplemental Tables 1–4. Supplemental Table 1 Exploratory analyses among POD patients. Supplemental Table 2 Factors significantly associated with postoperative delirium. Supplemental Table 3 Direct and indirect association between anti-inflammatory bundle and postoperative delirium. Supplemental Table 4 Direct and indirect association of anti-inflammatory bundle and alternate definition of the primary outcome.

Additional file 2. Figs. S1–S2. Fig. S1 Selection of predictors. Fig. S2 Preliminary mediation analysis.

## Data Availability

The datasets used and analyzed during the current study are available from the corresponding author on reasonable request.

## Abbreviations

POD: Postoperative delirium
NSAID: Nonsteroidal anti-inflammatory drug
CONSORT: Consolidated Standards of Reporting Trials
ASA: American Society of Anesthesiologists
THA: Total hip arthroplasty
PFNA: Proximal femoral nail anti-rotation
CAM: Confusion assessment method
RASS: Richmond Agitation-Sedation Scale
DRS-R-98: Delirium Rating Scale-Revised-98
NRS: Numeric rating scale
CSF: Cerebrospinal fluid
HADS: Hospital Anxiety and Depression Scale
MoCA: Montreal Cognitive Assessment
ICU: Intensive care unit
S100β: S100 calcium-binding protein β
NSE: Neuron-specific enolase
CRP: C-reactive protein
ANOVA: Analysis of variance
LASSO: Least absolute shrinkage and selection operator
