# Supplementary material for: Effect of combination of multiple anti-inflammatory drugs strategy on postoperative delirium among older patients undergoing hip fracture surgery: a pilot randomized controlled trial
Source: BMC Med. 2025 Feb 21;23:108. doi: 10.1186/s12916-025-03946-x (PMC11846162; doi:10.1186/s12916-025-03946-x)
Supplement: Supplementary file 1 — Additional file 1. Supplemental Tables 1–4. Supplemental Table 1 Exploratory analyses among POD patients. Supplemental Table 2 Factors significantly associated with postoperative delirium. Supplemental Table 3 Direct and indirect association between anti-inflammatory bundle and postoperative delirium. Supplemental Table 4 Direct and indirect association of anti-inflammatory bundle and alternate definition of the primary outcome. [file 12916_2025_3946_MOESM1_ESM.docx]

**Additional File 1: Supplemental Table 1-4**

**Supplemental Table 1.** Exploratory analyses among POD patients.

**Supplemental Table 2.** Factors significantly associated with postoperative delirium.

**Supplemental Table 3.** Direct and indirect association between anti-inflammatory bundle and postoperative delirium.

**Supplemental Table 4.** Direct and indirect association of anti-inflammatory bundle and alternate definition of the primary outcome.

**Supplemental Table 1.** **Exploratory analyses among POD patients.**

| **Outcomes** | **Anti-inflammatory bundle (*n* = 9)** | **Control group**  **(*n* = 27)** | **Risk ratio / Median difference** ***^a^* (95% CI)** | ***P* value** |
| --- | --- | --- | --- | --- |
| Motoric subtypes [*n* (%)] |  |  |  | 0.714 |
| Hyperactive | 3 (33.3) | 11 (40.7) | 0.82 (0.29, 2.29) |  |
| Hypoactive | 5 (55.6) | 15 (55.6) | 1.00 (0.51, 1.96) |  |
| Mixed | 1 (11.1) | 1 (3.7) | 3.00 (0.21, 43.20) |  |
| Severity score | 25 [18, 33] | 30 [30, 35] | -6 (-12, 0) | 0.045 |

*^a^* Pseudo-median difference was calculated using the Hodges-Lehmann estimate. Motoric subtypes were identified using the Richmond Agitation-Sedation Scale (RASS) and severity was measured using the Delirium Rating Scale-Revised-98 (DRS-R-98).

**Supplemental Table 2. Factors significantly associated with postoperative delirium.**

| **Variables** | **Without POD (*n* = 87)** | **With POD (*n* = 36)** | **OR (95%CI)** | ***P* value** |
| --- | --- | --- | --- | --- |
| **Group** |  |  |  |  |
| Anti-inflammatory bundle [*n* (%)] | 53 (61%) | 9 (25%) | 0.25 (0.07, 0.84) | 0.002 |
| **Baseline** |  |  |  |  |
| MoCA (IQR) | 26 [25, 27] | 25 [23, 26] | 0.62 (0.45, 0.83) | 0.028 |
| CRP [(IQR) mg L^-1^] | 7 [2, 35] | 40 [14, 76] | 1.01 (1.00, 1.03) | 0.002 |
| Hemoglobin (g L^-1^) | 114 ± 16 | 102 ± 18 | 0.97 (0.93, 1.00) | 0.044 |
| **Postoperative** |  |  |  |  |
| Pain at rest (NRS) | 1 [1, 2] | 2 [1, 3] | 1.83 (1.04, 3.33) | 0.048 |
| CRP [(IQR) mg L^-1^] | 60 [42, 84] | 115 [95, 139] | 1.02 (1.01, 1.03) | 0.040 |

The sample size may not be sufficiently powered to identify predictors for POD. POD, postoperative delirium; OR, odds ratio; MoCA, Montreal cognitive assessment; IQR, interquartile range; CRP, C-reactive protein; NRS, numeric rating scale.

| **Model** | **Direct effect**  **(OR, 95% CI)** | **Indirect effect via systemic inflammation *^b^* (OR, 95% CI) *^c^*** | **Indirect effect via postoperative pain**  **(OR, 95% CI) *^c^*** |
| --- | --- | --- | --- |
| **Model with only systemic inflammation** | | |  |
| Unadjusted | 0.32 (0.12, 0.83) *^a^* | 0.57 (0.26, 0.85) *^a^* | - |
| Adjusted *^d^* | 0.20 (0.06, 0.67) *^a^* | 0.61 (0.26, 0.87) *^a^* | - |
| **Model with only postoperative pain** | | | |
| Unadjusted | 0.31 (0.12, 0.77) *^a^* | - | 0.65 (0.41, 0.96) *^a^* |
| Adjusted *^d^* | 0.22 (0.07, 0.71) *^a^* | - | 0.64 (0.34, 1.01) |
| **Model with both systemic inflammation and postoperative pain** | | |  |
| Unadjusted | 0.43 (0.15, 1.23) | 0.57 (0.21, 0.88) *^a^* | 0.69 (0.40, 1.00) *^a^* |
| Adjusted *^d^* | 0.25 (0.07, 0.86) *^a^* | 0.64 (0.23, 0.89) *^a^* | 0.69 (0.36, 1.04) |

**Supplemental Table 3.** **Direct and indirect association between anti-inflammatory bundle and postoperative delirium.**

*^a^* These effects were considered statistically significant. *^b^* Systemic inflammation is indicated by C-reactive protein levels. *^c^* Bootstrapping (5000 iterations) was used to estimate confidence intervals. *^d^* Adjusted for baseline risk factors of postoperative delirium. OR, odds ratio; CI, confidence interval.

| **Model** | **Direct effect**  **(OR, 95% CI)** | **Indirect effect via systemic inflammation *^b^* (OR, 95% CI) *^c^*** | | **Indirect effect via postoperative pain**  **(OR, 95% CI) *^c^*** |
| --- | --- | --- | --- | --- |
| **Model with only systemic inflammation** | | |  | |
| Unadjusted | 0.23 (0.08, 0.63) *^a^* | 0.64 (0.32, 0.89) *^a^* | - | |
| Adjusted *^d^* | 0.14 (0.04, 0.49) *^a^* | 0.68 (0.33, 0.92) *^a^* | - | |
| **Model with only postoperative pain** | | | | |
| Unadjusted | 0.23 (0.09, 0.62) *^a^* | - | 0.69 (0.43, 1.01) | |
| Adjusted *^d^* | 0.15 (0.04, 0.53) *^a^* | - | 0.70 (0.39, 1.11) | |
| **Model with both systemic inflammation and postoperative pain** | | |  | |
| Unadjusted | 0.29 (0.10, 0.82) *^a^* | 0.65 (0.28, 0.91) *^a^* | 0.74 (0.45, 1.07) | |
| Adjusted *^d^* | 0.17 (0.05, 0.60) *^a^* | 0.70 (0.30, 0.94) *^a^* | 0.74 (0.41, 1.12) | |

**Supplemental Table 4.** **Direct and indirect association of anti-inflammatory bundle and alternate definition of the primary outcome.**

*^a^* These effects were considered statistically significant. *^b^* Systemic inflammation is indicated by C-reactive protein levels. *^c^* Bootstrapping (5000 iterations) was used to estimate confidence intervals. *^d^* Adjusted for baseline risk factors of postoperative delirium. OR, odds ratio; CI, confidence interval.
