# Supplementary material for: Effect of combination of multiple anti-inflammatory drugs strategy on postoperative delirium among older patients undergoing hip fracture surgery: a pilot randomized controlled trial
Source: BMC Med. 2025 Feb 21;23:108. doi: 10.1186/s12916-025-03946-x (PMC11846162; doi:10.1186/s12916-025-03946-x)
Supplement: Supplementary file 2 — Additional file 2. Figs. S1–S2. Fig. S1 Selection of predictors. Fig. S2 Preliminary mediation analysis. [file 12916_2025_3946_MOESM2_ESM.docx]

**Additional File 2: Fig. S1-S2**

**Fig. S1.** Selection of predictors.

**Fig. S2.** Preliminary Mediation analysis.

**
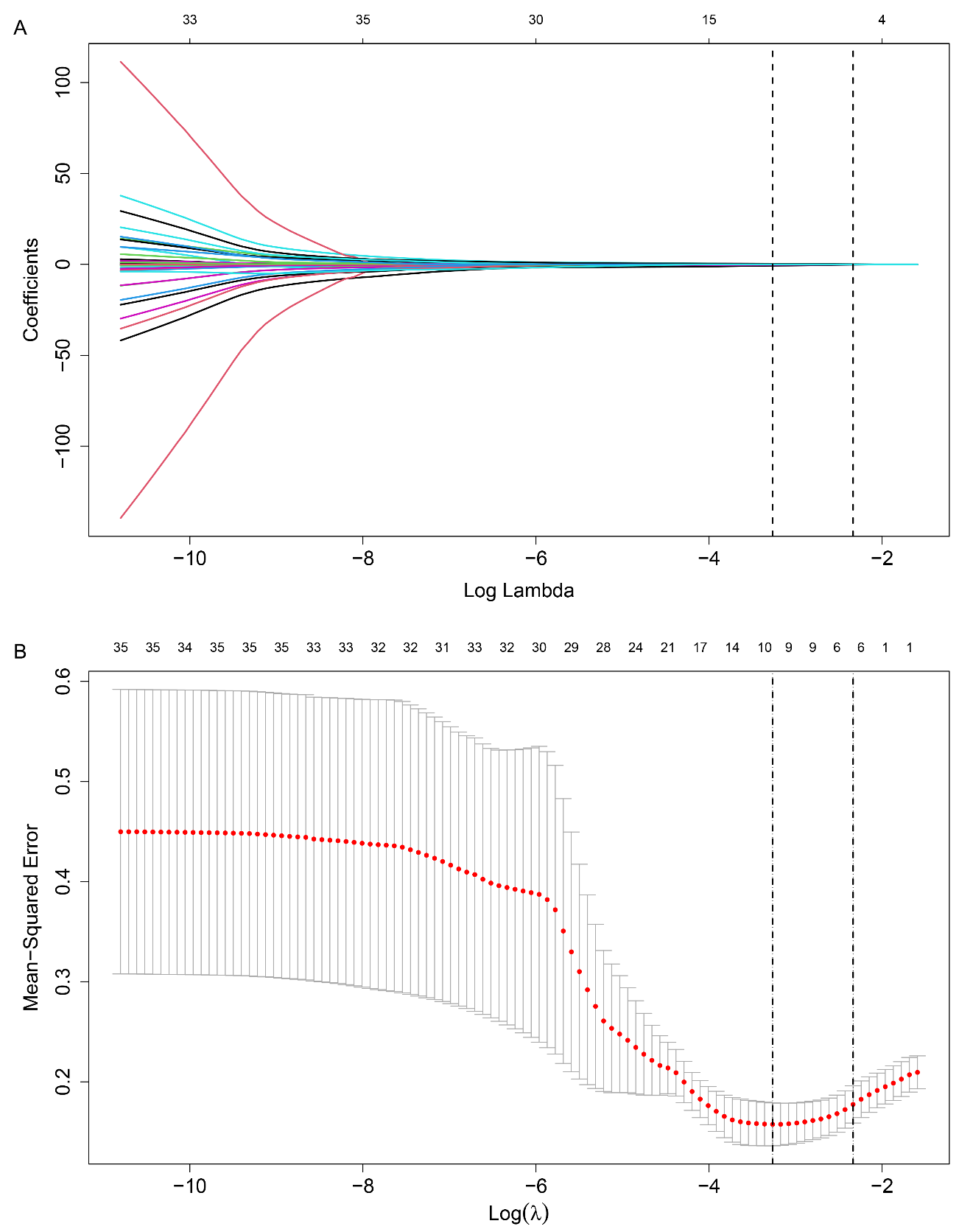
 Fig. S1. Selection of predictors.**

The selection was made using the least absolute shrinkage and selection operator (LASSO) regression analysis with seven-fold cross-validation. (A) The coefficient profile plot was generated against the log (lambda) sequence. (B) The tuning of the parameter (lambda) selection for deviance in LASSO regression was based on minimum and 1-standard error (SE) criteria (represented by the left and right dotted lines, respectively). In this study, the selection of predictors followed the 1-SE criterion, resulting in the choice of six nonzero predictors from the 34 coefficients.

**
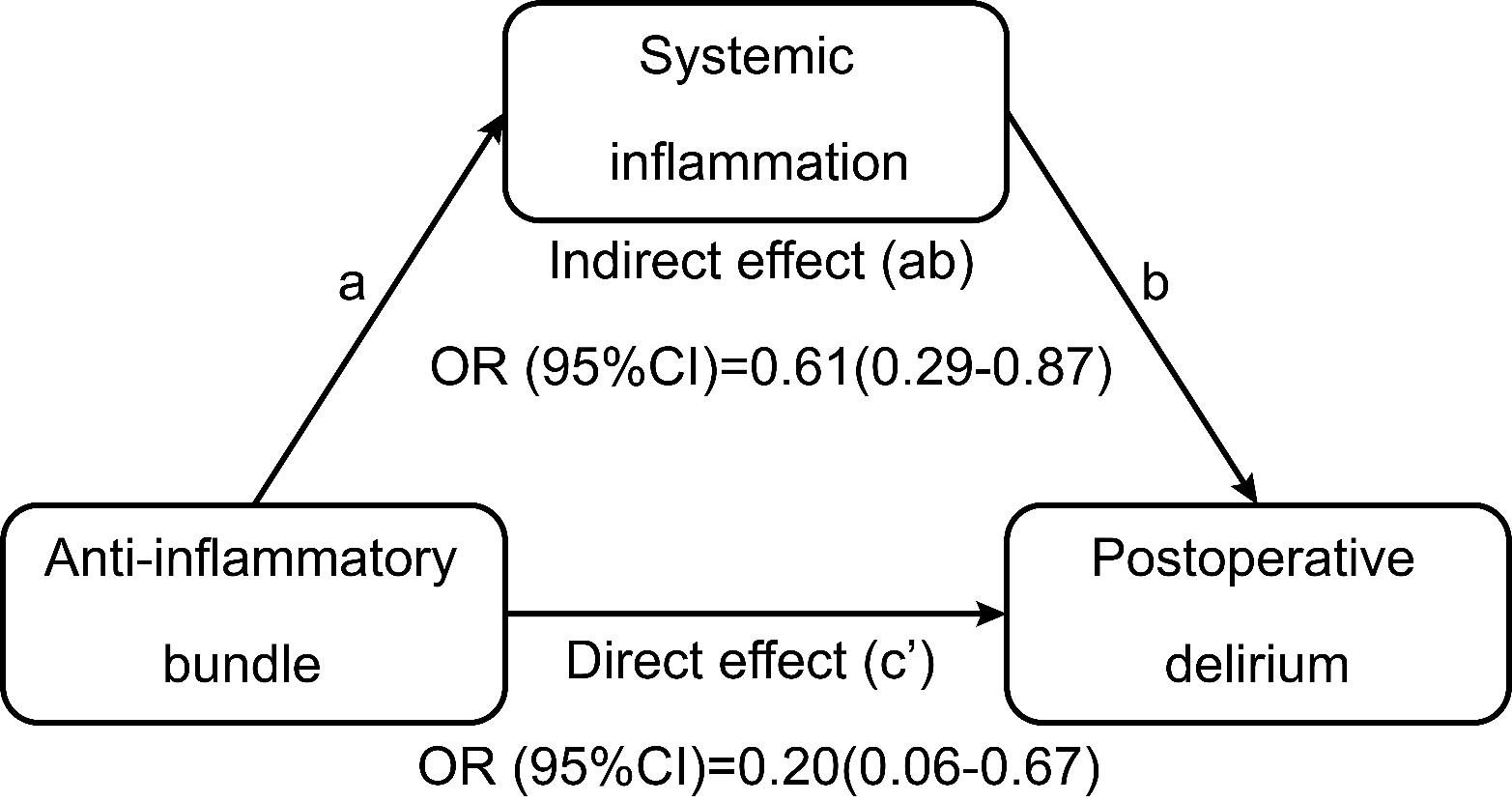
**

**Fig. S2.** **Preliminary mediation analysis.**

The direct effect of the anti-inflammatory bundle strategy on postoperative delirium, after accounting for the systemic inflammation level, is indicated by Path c’. The effect of the anti-inflammatory bundle strategy on the systemic inflammation level is represented by Path a. The effect of systemic inflammation on postoperative delirium, while adjusting for the anti-inflammatory bundle strategy, is represented by Path b. The indirect effect of the anti-inflammatory bundle strategy on postoperative delirium through systemic inflammation can be quantified as the product of Path a and Path b. OR, odds ratio; CI, confidence interval.
